# Successful Treatment of Infertility in a Patient with Probable 17 Hydroxylase Deficiency and Particularities of Association with Adrenal Autoimmunity—A Case Report and Review of the Literature

**DOI:** 10.3390/life13040921

**Published:** 2023-03-31

**Authors:** Alice Ioana Albu, Mirela Elena Iancu, Dragos Nicolae Albu

**Affiliations:** 1 Department of Endocrinology, Carol Davila University of Medicine and Pharmacy, 020021 Bucharest, Romania; 2 Endocrinology Department, Elias Hospital, 011461 Bucharest, Romania; 3 Endocrinology Department, ARTHOPE SRL, 077190 Bucharest, Romania; 4 Department of Obstetrics and Gynecology, Carol Davila University of Medicine and Pharmacy, 020021 Bucharest, Romania; dnalbu@gmail.com; 5 Reproductive Medicine Department, Medlife Hospital, 010719 Bucharest, Romania

**Keywords:** congenital adrenal hyperplasia, 17 hydroxylase deficiency, in vitro fertilization

## Abstract

Congenital adrenal hyperplasia (CAH) due to 17-hydroxylase deficiency (17OHD) is a rare disease accounting for less than 1% of cases of CAH. In female patients, fertility is severely affected mainly due to constantly increased progesterone affecting endometrium receptivity and implantation. The optimal treatment for infertility in these patients is not clearly established, with only a few recent case reports of successful pregnancies available in the literature. Hereby, we present the case of an infertile female patient with 17OHD who obtained pregnancy through an in vitro fertilization (IVF) freeze-all strategy and particularities of association with adrenal autoimmunity. A 32-year-old infertile female patient was referred for infertility evaluation and treatment. She had normal sex development and menstrual history with oligomenorrhea alternating with normal menstrual cycles. During the evaluation, a reduced ovarian reserve and obstruction of the left fallopian tube were identified, and IVF treatment was recommended. During a controlled ovarian stimulation for IVF, increased values of serum progesterone were observed; thus, all the embryos were frozen and additional tests were performed. Increased values of 17-hydroxyprogesteron, 11-deoxycorticosteron, and adrenocorticotropic hormones in association with low basal and stimulated serum cortisol, testosterone, androstenedione, and dehydroepiandrosterone sulfate were found, supporting the presence of 17OHD. She started treatment with oral hydrocortisone given at 20 mg/day but, because follicular phase serum progesterone remained high, hydrocortisone was replaced by an oral dexamethasone treatment of 0.5 mg/day, followed by the normalization of serum progesterone. A thawed blastocyst was transferred after preparation with oral estradiol at 6 mg/day and intravaginal progesterone at 600 mg/day under continuous suppression of endogenous progesterone production with a gonadotropin-releasing hormone agonist and oral dexamethasone. The patient became pregnant and delivered two healthy girls at term. One year after delivery, the presence of 21-hydroxylase antibodies was detected, which might explain the particularities of adrenal steroids in our patient. Our case report demonstrates that a patient with 17OHD can become pregnant through IVF and the transfer of thawed embryos in a subsequent cycle under continuous suppression of adrenal and ovarian progesterone production.

## 1. Introduction

Congenital adrenal hyperplasia (CAH) is a group of heterogeneous diseases with autosomal recessive inheritance, characterized by variate defects of the enzymes involved in adrenal steroidogenesis and a wide range of clinical manifestations from life-threatening salt-wasting to almost no symptoms [1]. The most frequent form of CAH is due to 21-hydroxylase deficiency (21OHD), which is responsible for 95% of all cases of CAH, while CAH due to 11-hydroxylase deficiency (11OHD) and 17-hydroxylase deficiency (17OHD) is encountered in less than 5% and 1% of cases, respectively [1]. The prevalence is variable, ranging from a relatively frequent disease in the nonclassical form of CAH due to 21OHD (1 in 200 in a Caucasian population) [1] to very rare disorders in 11OHD and 17OHD (1-9/1,000,000) [1]. Unusual for monogenic diseases, in CAH there is a lack of significant genotype–phenotype association [2] with a diverse clinical picture reflecting the changes in adrenal steroid production which, in turn, is proportional to the modification of enzyme activity. 17OHD is a rare form of CAH caused by mutations in the cytochrome P450 family 17 subfamily A member 1 (*CYP17A1*) gene located on chromosome 10q24.3. Mutation in this gene disrupts the activity of two enzymes located in both adrenals and gonads: 17alfa hydroxylase and 17, 20 lyase [3]. However, some mutations affecting *CYP17A1*, cytochrome P450 oxidoreductase (*POR*), or cytochrome B5 type A (*CYB5A*) genes may be responsible for the isolated 17, 20 lyase deficiency [3,4,5]. Most cases are described in consanguineous families [6] and some mutations are described in certain ethnic groups [6]. The severity of the disease is highly variable, with a milder presentation in case of mutations that retain partial catalytic activity, but even in patients with mutations leading to null enzyme activity, the severity and the age at the occurrence of symptoms may vary [6]. Environmental and genetic factors may be implicated in the modulation of the phenotypic features of patients with severe 17OHD [7]. It seems that the carriers of a heterozygous mutation in the *CYP17A1* gene do not present an obvious clinical picture, although they show reduced 17-hydroxylase activity and reduced adrenal reserve for steroid biosynthesis [8].

In patients with severe 17OHD, fertility is severely affected by a complex mechanism. Thus, decreased estrogen and androgen levels lead to arrested folliculogenesis and spermatogenesis, while constantly increased levels of progesterone during the follicular phase alter the endometrial receptivity, tubal motility, and cervical mucus thickness [5]. The optimal management of infertility in these patients is largely unknown, and most of the reported cases were not able to become pregnant with treatment [5]. It was suggested that the administration of hydrocortisone or more potent synthetic steroids, such as dexamethasone, may be useful for the suppression of progesterone production during infertility treatment [9]. The strategy of in vitro fertilization (IVF) with freeze-all embryos and transfers in a subsequent cycle is generally considered to avoid controlled ovarian stimulation-induced increased progesterone [9]. Only a few recent case reports of successfully treated infertile patients with 17OHD are available in the literature.

Hereby, we present the case of a young infertile woman with a diagnosis of CAH due to 17OHD who became pregnant through IVF and gave birth to two healthy girls. Moreover, clinical and hormonal particularities due to association with adrenal autoimmunity are discussed.

## 2. Case Report

**Figure 1:**
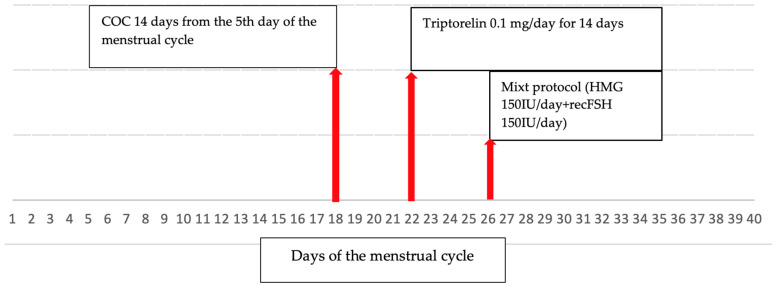
Controlled ovarian stimulation protocol for in vitro fertilization. COC, combined oral contraceptives; HMG, human menopausal gonadotropin; recFSH, recombinant follicle-stimulating hormone.

We present the case of a 32-year-old Caucasian woman, referred for infertility evaluation and treatment to the Reproductive Department of a private hospital. She had two previous intrauterine inseminations at another Center, without pregnancy. The evaluation of the couple revealed a decreased AMH value (0.74 ng/mL) and at transvaginal ultrasound a normal uterine aspect with proliferative endometrium and an antral follicle count of 9. A dominant follicle was also observed on the 12th day of the menstrual cycle. A positive mixed antiglobulin reaction (MAR) test was found in the male partner. Tubal patency was checked with histerosonography and an obstruction of the left fallopian tube was observed. After the evaluation, the diagnosis of primary infertility due to mixt causes was established (decreased ovarian reserve, male factor, and tubal obstruction), and an IVF procedure was recommended. She started treatment with a microflare protocol consisting of combined oral contraceptive (COC) administration for 14 days starting on the 5th day of the menstrual cycle, followed by triptorelin at 0.1 mg/day starting on the 4th day after COC interruption, for 14 days, and a mixt protocol for controlled ovarian stimulation (COS) starting in the 8th day after COC interruption consisting of the administration of 150 IU of human menopausal gonadotropin (HMG) in association with 150 IU of follitropinum alfa, which was taken daily for 10 days (Figure 1). Transvaginal egg collection was performed 36 h after the administration of 250 mg of recombinant human chorionic gonadotropin (HCG). Seven eggs were collected and, after classical IVF, we obtained five zygotes. On day three, we obtained three good embryos, two embryos with eight cells of grade 1 and one embryo with eight cells of grade 1.5, which evolved in a very good quality blastocyst 4aa on day five, and the other two arrested in evolution. The embryos’ quality was assessed according to Gardner–Schoolcraft criteria and Alpha Scientists in Reproductive Medicine and ESHRE Special Interest Group of Embryology criteria [10]. During COS, high serum progesterone levels with values of 4.3–6.2 ng/mL were observed. Because serum progesterone levels during COS exceeding 1.5 ng/mL is considered a poor prognosis factor for clinical pregnancy [11], the blastocyst was frozen, with a planned transfer in a subsequent cycle. High serum progesterone levels during COS raised the suspicion of CAH; thus, an early follicular phase serum 17-hydroxyprogesterone was measured in the morning and increased values were obtained (15.6 ng/mL, normal range 0.1–0.8 ng/mL). Therefore, the patient was referred for endocrinological evaluation.

At the endocrinological exam, we noticed a normal body mass index of 21 kg/m^2^, normal blood pressure without postural hypotension, and only a mild generalized pigmentation of the skin, which was considered the consequence of recent sun exposure according to the patient’s statement. Moreover, a palpable and elastic thyroid gland was identified. No other remarkable signs and symptoms were noted. She had no significant medical family history. Her menstrual history included menarche at the age of 14 and periods of oligomenorrhea (menstrual cycles of 40–50 days) alternating with regular menstrual cycles throughout her life, and she had no previous pregnancies. Her medical history included only autoimmune thyroiditis with hypothyroidism under treatment with 50 micrograms of Levo-thyroxin daily.

Laboratory evaluation (Table 1) confirmed the high value of early morning serum 17-hydroxyprogesterone level measured in the early follicular phase (12.3 ng/mL, normal range 0.1–0.8 ng/mL) in association with a low normal morning serum cortisol of 5.68 micrograms/dL (normal range 5–25 micrograms/dL) and high adrenocorticotropic hormone (ACTH) levels of >2000 pg/mL (normal range 7.2–63.3 pg/mL). Total testosterone was 6.38 ng/mL (normal range 8.4–48.31 ng/mL), dehydroepiandrosterone sulfate was low (6.3 microg/dL, normal range 98.8–340), and 11-deoxycorticosterone 0.26 microg/L was slightly increased (normal range <0.16 microg/L). The serum aldosterone level was 1.92 ng/dL (normal range 2.52–39.2 ng/dL) and the plasma direct renin level was 2 microIU/mL (normal range 4.4–46.1 microIU/mL). The serum level of the follicle-stimulating hormone (FSH) was 7.42 mIU/mL (normal range 1.5–11.7 mUI/mL), the luteinizing hormone (LH) was 8.32 mIU/mL (normal range 1.7–15 mUI/mL), and estradiol was 144.4 pg/mL (normal range 30–110 pg/mL). We also found low serum sodium of 130 mmol/L (normal range 136–145 mmol/L) and normal serum potassium of 4.6 mmol/L (3.5–5.1 mmol/L). Thyroid tests were normal, with a serum thyroid stimulating hormone (TSH) of 2.49 microIU/mL (normal range 0.4–4 microIU/mL) and serum free thyroxine of 1.44 ng/dL (normal range 0.89–1.76 ng/dL). A thyroid ultrasound revealed a normal dimension of the thyroid with an inhomogeneous structure, highly suggestive of autoimmune thyroiditis.

A 250 microgram synthetic ACTH (tetracosactide) stimulation test was performed which showed unstimulated cortisol of 5.35 micrograms/dl and 17-hydroxyprogesterone (12.92 ng/mL) levels and only slightly increased 11-deoxycorticosterone in comparison with the baseline of 0.36 microg/L one hour after stimulation (Table 1), which was probably due to already high endogenous ACTH levels.

Based on the hormonal profile with low serum cortisol, low androgens, and high 17 hydroxyprogesterone and 11 deoxycorticosterone, we suspected a 17-hydroxylase deficiency. Genetic testing for CAH was recommended but the patient refused for the moment. The patient started treatment with oral hydrocortisone in doses of 25 mg/day.

Computed tomography of the adrenal glands was performed and two nodular lesions of 5/5 mm and 9/6 mm were shown in the right adrenal gland, with an aspect highly suggestive of an adenoma.

After two months of hydrocortisone acetate treatment, her ACTH values declined to 370 pg/mL, but serum progesterone remained high (1.8 ng/mL, normal range 0.15–0.8 ng/mL) (Table 1). The hydrocortisone was replaced with dexamethasone 0.75 mg/day and serum progesterone became normal (0.21 ng/mL in the early follicular phase) (Table 1). The patient started preparation for frozen embryo transfer with a triptorelin administration of 3.75 mg for further suppression of gonadotropins on day 21 of the previous cycle, before the transfer of cryopreserved blastocyst. We started the endometrium preparation on the second day of the menstrual cycle with estradiol valerate administration of 2 mg three times daily. On the 12th day of estradiol administration, the endometrium thickness was 9 mm, and an intravaginal progesterone administration of 200 mg three times a day was associated. After five days of progesterone administration, the thawed blastocyst was transferred. The dexamethasone treatment was maintained until two days before embryo transfer, and then was replaced with oral hydrocortisone at 25 mg/day. Luteal phase support was provided using estradiol valerate, 2 mg three times per day, and intravaginal progesterone, 200 mg three times per day.

The measurement of serum ßHCG 12 days after embryo transfer showed a value over 14 UI/L, which was considered diagnostic for pregnancy. Clinical pregnancy was confirmed by ultrasound visualization of a twin intrauterine pregnancy with a fetal heartbeat. The treatment with estradiol valerate, 2 mg 3 times per day, and intravaginally progesterone, 200 mg three times per day, was continued until the 12 weeks of pregnancy, and oral hydrocortisone administration of 25 mg/day was continued throughout the pregnancy. The patient decided to perform the genetic analysis in the first trimester of pregnancy and a commercial next-generation sequencing (NGS) panel for multiple genes involved in CAH was performed but did not identify mutations in *ARMC5, CYP11A1, CYP11B1, CYP11B2, CYP17A1, CYP21A2, HSD3B2, PDE11A, PDE8B, POR, PRKAR1A*, and *STAR.* The patient delivered two healthy girls at term.

One year after the delivery, the patient underwent a routine reevaluation. She continued the treatment with hydrocortisone at 25 mg/day. Her blood pressure was normal and her menstrual cycles became regular after delivery. Adrenal tomography was repeated and showed atrophic adrenal glands. Considering the presence of autoimmune thyroiditis and the evolution of adrenal imaging, we decided to measure the 21hydroxilase antibodies which were found to be increased (66.3 antibodies ratio, normal range <10), confirming the presence of adrenal autoimmunity.

## 3. Discussion

The adrenal steroid profile in our patient with high progesterone, 11-deoxycorticosterone, and low cortisol and androgens suggests the presence of 17OHD. Probably, the presence of adrenal autoimmunity modified the adrenal steroidogenesis by decreasing the levels of the adrenal hormones and, therefore, was a confounding factor for the severity of the enzyme deficiency.

The association of 17OHD with adrenal autoimmunity was not previously reported. However, a case of autoimmune primary adrenal insufficiency in a patient with 21-hydroxylase deficiency seeking infertility treatment was described, with menstrual abnormalities becoming evident at the age of 22 [12]. The authors hypothesized that the presence of adrenal insufficiency and high ACTH levels stimulated the adrenal glands and aggravated the adrenal hormone abnormalities caused by an enzyme deficiency, contributing to the diagnosis of gene defects and the infertility of the patient [12]. The same might be also true in the case of our patient when the significantly increased ACTH levels probably contributed to unmasking a mild enzyme defect.

17OHD was described for the first time in 1966 by Biglieri et al. [13]. Since then, more than 100 mutations have been reported in the *CYP17A1* gene, including point mutations, small deletions or insertions, splice-site alterations and, less frequent, large deletions [14]. Most of these mutations are associated with the classical phenotype of the disease, with only a small number of *CYP17A1* missense variants being reported to impair only partially the activity of 17α-hydroxylase/17, 20-lyase activity [15]. Because of the variate degree attenuation of enzymatic activity of 17α-hydroxylase and 17, 20-lyase, the complete or partial deficiency of the two enzymes can occur [14]. The resulting abnormalities of adrenal steroidogenesis are reflected in the clinical picture.

In patients with a complete deficiency of 17 hydroxylase/17, 20-lyase activity, androgen and estrogen production decreased. Cortisol production is also decreased, but high levels of corticosterone compensate for its glucocorticoid activity. In turn, both corticosterone and deoxycorticosterone excess, by their mineralocorticoid activity, contribute to plasma volume expansion and suppresses plasma renin activity. Progesterone also accumulates above the block at 17-hydroxylase, and high progesterone is found in 17OHD particularly in POR [5]. Because of these hormonal abnormalities, the classical clinical phenotype of patients with complete 17OHD includes symptoms of hypergonadotropic hypogonadism with a lack of pubertal development and primary amenorrhea in girls [16,17] and female external genitalia in boys with a blind vaginal pouch [5]. The increased levels of products with mineralocorticoid activity cause high blood pressure and hypokalemia. Most of the cases reported in the literature are in 46XY patients, with the diagnosis in 46XX patients being established with more difficulty due to the rarity of the disease as a cause of hypergonadotropic hypogonadism. However, these clinical manifestations are not constantly found in all the 46XX patients, with only 71% of patients presenting primary amenorrhea, 50% with no breast development, and 88% with high blood pressure, as reported in a case series of sixteen female patients with CAH due to inactivating *CYP17A1* mutations [18]. There are few case reports of 46XX patients with *CYP17A1* defects in the literature and the knowledge about this form of the disease continues to increase, and new phenotypes are probably to be reported. In patients with partial 17OHD, a milder clinical picture was described, including the development of secondary sexual characteristics and mild or even absent hypertension [14,19,20]. Female patients with only infertility as a clinical manifestation [21,22] and boys with only hypospadias and micropenis [14] were also described. In general, patients with over 25% of residual enzyme activity might have no signs suggestive of diagnosis [8].

The clinical picture of our patient was completely unremarkable, without any clinical sign suggestive of 17OHD. The patient had normal pubertal progression and the menstrual cycles were only slightly irregular, the blood pressure was normal, and the body hair was normally represented. At the first evaluation, the main cause of infertility was considered the decreased ovarian reserve and the only suspicious finding was significantly increased serum progesterone levels during controlled ovarian stimulation, which prompted the measurement of serum 17hydroxyprogesteron. In the absence of a desire for pregnancy, there is an increased probability that the diagnosis, in this case, would not be established.

Previous reports of 17OHD patients suggested that some findings could be a trait of the disease. Thus, adrenal adenoma, also present in our patient, was considered a feature of 17OHD in a case series of sixteen patients with 46XX karyotype [18] and one case report [17]. The evolution of adrenal imaging with the disappearance of adrenal adenoma over time in our patient is probably a consequence of adrenal autoimmunity and the destruction of the adrenal glands. Similarly, recurrent ovarian cysts were reported as a feature of 17OHD by some authors [15,18], while others found streak gonads [5,22]. However, in our patient, no history of ovarian cysts was present, and no ovarian cyst formation was noticed during the follow-up period. A possible explanation is the increased level of gonadotropins in the previous studies which continuously stimulate the ovaries and determine the formation of cysts. In contrast, in our patient the LH and FSH levels were normal.

The increased values of morning serum levels of 17-hydroxyprogesterone observed in our patient are not typically described in patients with 17OHD. Especially in cases with severely decreased or abolished 17 hydroxilase activity, the decreased serum cortisol levels are associated with normal or even decreased serum 17-hydroxyprogesterone levels [18]. However, there are few case reports with low cortisol and high 17-hydroxyprogesterone, usually in patients with residual 17 hydroxylase activity [14,15,18].

Although most of the reports of patients with 17OHD showed a decreased level of circulating estrogens [5], in our patient, estrogens were normal. Few other patients with normal estrogens were reported [21]. Some of the authors consider the normal estrogen level as a consequence of an alternate (‘backdoor’) pathway for steroid production, which is compensating in these patients for the enzyme blockage in the 17hydroxylase activity [23].

Fertility is severely compromised in complete 17 OHD deficiency, and no cases of spontaneous pregnancy were described in the literature, but there are a few cases of individuals who achieved pregnancy with the use of assisted reproductive techniques. An early report by Levran et al. [21] described in detail the hormonal profile of four patients with combined partial 17-hydroxylase/17, 20 lyase deficiency, and who were phenotypically normal. They presented only primary infertility, anovulation, and persistent alteration of the cervical mucus [21]. The authors showed that the administration of the gonadotropin-releasing hormone (GnRH) agonist triptorelin at 0.5 mg/day in association with dexamethasone (0.5 mg/day orally starting on the 14th day of triptorelin administration) can reduce the progesterone production, followed by the achievement of normal follicular phase serum progesterone [21]. However, the administration of HMG for ovarian stimulation before IVF resulted in abnormally high follicular phase progesterone levels. The number of oocytes obtained was between two and thirty-four, with a fertilization rate of 50–91%. The transfer of fresh embryos did not result in pregnancy in any of the four patients. Thus, another attempt at IVF in one of the patients with COS under gonadal and adrenal suppression with dexamethasone and a GnRH agonist was performed, and all the resulting embryos were frozen. The subsequent transfer of thawed embryos after preparation of the endometrium with exogenous estrogen and progesterone administration under the same suppression regimen was, however, followed by pregnancy and the live birth of three triplets [21].

In 2016, Bianchi reported the case of a 29-year-old 46XX patient with 17OHD due to a compound heterozygous mutation (p.W406R/P428L) in the *CYP17A1* gene with the desire to become pregnant [4]. Her clinical picture included primary amenorrhea and high blood pressure. She was under treatment with oral dexamethasone of 0.5 mg/day and hormonal replacement with conjugated equine estrogen (CEE) of 0.625 mg/day. She had a previous unsuccessful IVF attempt and started preparation for another IVF cycle with oral dexamethasone acetate of 0.5 mg/day and oral estradiol valerate of 4 mg/day, followed, after 12 days, by natural micronized progesterone of 200 mg/day administrated vaginally. Beginning on the 19th day of the estrogen administration, leuprolide acetate, a GnRH agonist, of 0.5 mg/day was administered subcutaneously to achieve downregulation of gonadotropin production. At the interruption of the estrogen and progesterone administration, menstrual bleeding occurred, and the patient started COS with recombinant FSH 112.5 U/day associated with oral CEE at 0.625 mg/day under continuous dexamethasone suppression (0.5 mg/day). At the end of the COS four follicles ≥15 mm were obtained, but serum estradiol was low (<13 pg/mL) and serum progesterone increased (13.1 ng/mL). Four mature oocytes were retrieved and two blastocysts were obtained, which were cryopreserved due to high progesterone levels. One month after oocyte retrieval, the patient received associated treatment with a GnRH agonist (depot goserelin acetate) and oral dexamethasone of 0.5 mg/day to control both the ovarian and adrenal progesterone overproduction. Three months later, serum progesterone was 0.6 ng/mL, and endometrial preparation was started with oral estradiol valerate at 6 mg/day in association with natural micronized progesterone at 600 mg/day administered intravaginally. Two thawed embryos were transferred after 5 days of progesterone administration and β-hCG tested positive 14 days later. Vaginal progesterone supplementation and oral dexamethasone acetate at 0.5 mg/day were maintained by the end of the pregnancy. The pregnancy was complicated by pre-eclampsia and gestational diabetes and the patient gave birth to a normal male newborn at 30 weeks and 4 days of gestation due to acute fetal distress.

A case report of a *CYP17A1* gene mutation caused by homozygous deletion of exon 1–6 causing hypertension, hypokalemia, decreased cortisol levels [22], and infertility in a 39-year-old woman was described. Her menstrual history was unremarkable, with menarche at 12 years of age and regular menses. Pregnancy was obtained in the second IVF cycle at the age of 43, despite the reported presence of streak gonads [22]. IVF treatment was performed in association with prednisone administration at a dose of 7.5 mg/day, but no other details of the IVF protocol or hormone levels during stimulation were provided [22].

Offering a more thorough description of the assisted reproductive techniques used, Kitajima et al. presented a case of a 24-year-old female with a homozygous deletion in exon 1 with primary amenorrhea and hypertension who wished to become pregnant [24]. In vitro fertilization with COS was first performed, with a nasal GnRH agonist (buserelin acetate) and daily administration of pure FSH 150 IU, followed by 10,000 IU HCG trigger. Serum progesterone levels were markedly increased during COS. Thus, the 21 oocytes retrieved were fertilized by conventional IVF, and 12 blastocysts were obtained and cryopreserved. Three months after blastocyst freezing, endometrium preparation was peformed with a transdermal estradiol patch and an intramuscular progesterone injection of 50 mg daily under glucocorticoid administration. Two transfers of thawed embryos were performed 2.5 years apart followed by pregnancies and the deliveries of two healthy babies [24].

Recently, Blumfeldt et al. [25] described the case of a patient with 17, 20 lyase deficiency due to a p.E305G mutation in exon 5 of the *CYP17A1* gene, who became pregnant through IVF followed by frozen-thawed embryo transfer. The patient had a hormonal profile characterized by low androgen and estrogen, low basal and stimulated cortisol levels, and high follicular phase serum progesterone. She had three previous unsuccessful IVF attempts with the retrieval of oocytes and fertilizations, but no conceptions. In the new IVF cycle, downregulated with the long GnRH agonist protocol, she received a treatment of 30 mg of prednisone daily, and HMG for controlled ovarian stimulation for 11 days [25]. Thirty-six hours after the administration of 250 µg of recombinant HCG for follicular maturation, 37 oocytes were retrieved. After intracytoplasmic sperm injection, 25 ova were fertilized, and 17 embryos were cryopreserved. Due to the high serum progesterone concentration, no fresh embryo transfer was carried out and all embryos were cryopreserved. Two embryos were transferred after preparation of the endometrium with 10 mg of daily oral 17β estradiol for 11 days under progesterone suppression with a long-acting GnRH agonist and prednisone taken at 30 mg/day. When an endometrial width of 8.5 mm was reached, vaginal progesterone was added at 100 mg three times/day for four days before embryo transfer. Pregnancy was confirmed by ultrasound, but it ended in missed abortion. After 2 months, still under the suppression of the long-acting GnRH agonist and oral prednisone, a similar endometrial preparation was repeated using oral estradiol and vaginal progesterone. Two embryos were thawed and transferred, and pregnancy was confirmed by ultrasound three weeks later. Daily prednisone treatment was gradually decreased to 20 and then 10 mg/day and was continued at a dose of 5 mg/day throughout pregnancy. The patient gave birth to a normal female neonate at 41 weeks of gestation [25].

Other authors failed to obtain pregnancy. Rabinovici et al. treated a 30-year-old female with IVF and 17OHD was diagnosed based on the hormonal profile [26]. Prednisone and GnRH agonist treatment were administrated for the suppression of progesterone production, and urofollitropin was administrated for ovarian stimulation. During stimulation, plasma estradiol was undetectable, but at egg collection three oocytes were obtained and two embryos developed until the in-cleavage stage with seven cells, when they were arrested. Therefore, no embryo transfer was possible [26].

Another report of a 31-year-old female with primary amenorrhea and infertility, diagnosed with 17OHD based on the hormonal profile of ovarian follicular fluid and granulosa-luteal cells [27], resulted in no pregnancy. She received ovarian stimulation with pure FSH and HCG for oocyte maturation, and 19 oocytes were retrieved, of which 11 fertilized. Four fresh embryos were transferred and the remaining were cryopreserved. As no pregnancy was obtained, four thawed embryos were transferred, also without pregnancy. The authors concluded that oocyte maturation and fertilization are not impaired in patients with 17OHD despite insufficient estradiol levels.

Another report of a 28-year-old female with 17OHD, also diagnosed by the hormonal profile, treated with two cycles of controlled ovarian stimulation and IUI followed by a cycle of IVF [28], resulted in no pregnancy. Before ovarian stimulation, for IUI the patient was treated with testosterone propionate administrated by laparoscopic intraovarian injection to overcome the arrested folliculogenesis due to a lack of androgens. Although one or two follicles over 18 mm were obtained, no pregnancy was achieved. The treatment was followed by an IVF cycle with testosterone administration by vaginal suppositories in association with high doses of exogenous gonadotropins. Six oocytes were collected, but none fertilized probably due to their abnormal appearance (intracytoplasmic vacuoles and thickened zona pellucida).

Matsuzaki et al. reported the case of a 26-year-old patient with secondary amenorrhea and infertility, who was a compound heterozygote for delF53/54 and the missense mutation H373L [20]. She was treated with oral dexamethasone of 0.5 mg/day, transdermal estradiol, and intramuscular progesterone for endometrial preparation and recombinant FSH for ovulation induction. One ovarian follicle of 19 mm was obtained, which resulted in no pregnancy. The endometrium remained below 6 mm thick during treatment. Repeated histologic evaluation performed during progesterone administration showed unripe endometrium with glands in the early secretory phase and markedly scanty stroma, with no reactivity for estrogen receptors in stromal cells. Progesterone levels remained high during treatment.

The data from the literature and our data suggest that GnRH agonists and oral glucocorticoids are not enough for the adequate suppression of progesterone production during COS, and higher levels are a probable cause of decreased endometrial receptivity and IVF failure in fresh cycles. Thus, cryopreservation of the embryos and the transfer of thawed embryos in a subsequent cycle seems to be the best option for IVF success. Moreover, for best results of the endometrial preparation, the suppression of ovarian and adrenal progesterone production should be continued with the GnRH agonist and supraphysiologic doses of exogenous glucocorticoids. Although considered a cause of arrested folliculogenesis and a contributor to infertility, decreased testosterone level is not a major cause of IVF failure, and its correction is not necessary for adequate folliculogenesis [28]. Despite increased progesterone and decreased estrogen levels during COS, which was suggested as deleterious for oocytes fertilization [29], some of the reported patients obtained a high number of oocytes that fertilized normally, supporting the idea that these hormonal imbalances are not the main cause of IVF failure in 17OHD.

Because the molecular diagnosis was negative in our patient, we took into consideration the differential diagnosis and other causes of elevated 17-OH progesterone. There have been case reports in the literature where a 17-hydroxyprogesterone-secreting adrenal tumor mimicked the clinical milieu of CAH, but suppression after dexamethasone was not observed and imaging techniques clarified the diagnosis [30]. Furthermore, ovarian steroid cell tumors may present with increased 17-hydroxyprogesterone levels and may sometimes show a similar profile as CAH after ACTH stimulation. However, a different clinical and hormonal picture than in the actual case report is present and the primary tumor is generally visible [31]. Although autoimmune primary adrenal insufficiency is a cause of decreased cortisol and adrenal androgens production, the disproportionately increased 17-hydroxyprogesterone and 11-deoxycorticosterone levels found in our patient suggest that another entity is causing the adrenal steroidogenesis imbalance. Thus, using a low-dose ACTH stimulation test (1 μg) for the diagnosis of preclinical autoimmune adrenal insufficiency, Laureti et al. were able to demonstrate that dehydroepiandrosterone sulfate, 17-hydroxyprogesterone, and aldosterone correlate with the cortisol levels and that the autoimmune process causes decreased secretion of all adrenocortical hormones, even in the subclinical stage of the disease [32].

A possible explanation for the failure of NGS analysis performed on our patient to detect any anomaly in the *CYP17A1* gene is the presence of a new mutation causing 17OHD. Moreover, NGS was not performed for the *CYB5A* gene, which was reported to be involved in 17, 20 lyase deficiency. However, methemoglobinemia seems to be a constant trait of *CYB5A* gene mutations causing 17, 20 lyase deficiency [33,34,35]. The presence of methemoglobinemia is improbable in our patient due to the lack of any suggestive clinical signs, although specific tests to identify methemoglobinemia were not performed. Previous reports of *CYB5A* causing 17, 20 lyase deficiency described decreased androgens, normal cortisol and, inconstantly, mildly elevated 17-hydroxyprogesterone levels [33,34,35]. Although the serum cortisol level decreased in our patient, it could be due to adrenal autoimmunity and not to adrenal steroidogenesis defects.

## 4. Conclusions

Our case report supports the idea that infertile patients with 17OHD can become pregnant with IVF followed by a freeze-all strategy and transfer of the thawed embryos in a subsequent cycle under continuous suppression of the progesterone production with dexamethasone and a GnRH agonist. Patients with 17OHD can be completely asymptomatic before attempting to become pregnant and this diagnosis should be kept in mind in a patient with high progesterone levels during COS.

## Figures and Tables

**Table 1:** Evolution of hormonal parameters under glucocorticoid treatment.

Serum Hormone Levels	Normal Range	Value at Diagnosis	Values 1 h after a Short Synacthen Test	After 2 Months of Hydrocortisone Treatment at 25 mg/Day	After Dexamethasone Treatment at 0.75 mg/Day
Cortisol (μg/dL)	5–25	5.68	5.35	0.4	<0.05
ACTH (pg/mL)	7.2–63.3	>2000		370	1.27
17-hydroxyprogesterone (ng/mL)	0.1–0.8	12.3	12.92		
Progesterone (ng/mL)	0.15–0.8	6.4		1.8	0.21
11-deoxycorticosterone (μg/L)	<0.16	0.26	0.36		
Total testosterone (ng/mL)	8.4–48.31	6.38			
DHEAS (μg/dL)	98.8–340	6.3			

ACTH, adrenocorticotropic hormone; DHEAS, dehydroepiandrosterone sulphate.

## Data Availability

Not applicable.
